# Hemophagocytic lymphohistiocytosis in gastric cancer: A rare syndrome for the oncologist. Case report and brief review

**DOI:** 10.3389/fonc.2023.1010561

**Published:** 2023-02-08

**Authors:** Manlio Monti, Giovanni Marconi, Andrea Ambrosini-Spaltro, Chiara Gallio, Virginia Ghini, Luca Esposito, Stefano Antonini, Daniela Montanari, Giovanni Luca Frassineti

**Affiliations:** ^1^ Department of Medical Oncology, IRCCS Istituto Romagnolo per lo Studio dei Tumori (IRST) “Dino Amadori”, Meldola, Italy; ^2^ Haematology Unit, IRCCS Istituto Romagnolo per lo Studio dei Tumori (IRST) “Dino Amadori”, Meldola, Italy; ^3^ Pathology Unit, “Morgagni-Pierantoni” Hospital, AUSL della Romagna, Forlì, Italy

**Keywords:** hemophagocytic lymphohistiocytosis, gastric cancer, secondary hemophagocytic lymphohistiocytosis, disseminated intravascular coagulation, case report

## Abstract

Hemophagocytic lymphohistiocytosis (HLH) is a rare and life-threatening condition characterized by uncontrolled activation of the immune system. HLH is a reactive mononuclear phagocytic response that occurs in association with a constellation of conditions such as malignancies and infections. The clinical diagnosis of HLH remains challenging because HLH can present with symptoms that significantly overlap with other causes of cytopenia, such as sepsis, autoimmune diseases, hematological cancers, and multiorgan failure. A 50-year-old man went to the emergency room (ER) for hyperchromic urine, melena, gingivorrhagia, and spontaneous abdominal wall hematomas. The first blood tests showed severe thrombocytopenia, alteration of the INR, and consumption of fibrinogen, and therefore, a diagnosis of disseminated intravascular coagulation (DIC) was made. A bone marrow aspirate showed numerous images of hemophagocytosis. With the suspicion of immune-mediated cytopenia, oral etoposide, intravenous immunoglobulin, and intravenous methylprednisolone were administered. Then, a diagnosis of gastric carcinoma was performed with a lymph node biopsy and gastroscopy. On the 30th day, the patient was transferred to the oncology ward of another hospital. On admission, he had serious piastrinopenia, anemia, hypertriglyceridemia, and hyperferritinemia. He was supported with a platelet transfusion and underwent a bone biopsy that showed a picture compatible with myelophthisis from diffuse medullary localization of a carcinoma of gastric origin. A diagnosis of HLH secondary to solid neoplasm was formulated. The patient started chemotherapy with oxaliplatin, calcium levofolinate, 5-fluorouracil bolus, 5-fluorouracil for 48 h (mFOLFOX6), and methylprednisolone. Six days after the third cycle of mFOLFOX6, the patient was discharged with the stabilization of his piastrinopenia condition. The patient continued chemotherapy with an improvement in his clinical conditions and normalization of hematological values. After 12 cycles of mFOLFOX, it was decided to start maintenance chemotherapy with capecitabine but, unfortunately, after only one cycle, HLH reappeared. The oncologist has to keep in mind the existence of HLH when there is an unusual clinical presentation of cancer, such as cytopenia affecting ≥2 lineages and alterations of ferritin and triglycerides other than fibrinogen and coagulation. Increased attention and additional research as well as a close collaboration with hematologists are needed to benefit patients with solid tumors complicated by HLH.

## Introduction

Hemophagocytic lymphohistiocytosis (HLH) is a rare and life-threatening condition characterized by uncontrolled activation of the immune system (1, 2).

HLH was first described in 1939 by Scott and Robb-Smith and again in 1952 when Farquhar and Claireaux reported a case of two infant siblings with progressive and fatal cytopenias, hepatosplenomegaly, and fever with the autopsy showing hemophagocytosis (3, 4). HLH has an estimated annual incidence of 1 per 800,000 people in Japan (5) and less than 10 per 1,000,000 children in Italy, Sweden, and the United States (6–8).

HLH can be classified into primary and secondary. Primary HLH is an inherited, autosomal recessive disorder associated with defects in perforin function. Perforin is a cytolytic protein found in the granules of cytotoxic T lymphocytes and natural killer cells. The degranulation of these leukocytes releases perforin, which inserts itself into the cell membrane of the target cell, creating a pore causing cell lysis and subsequently an inflammatory storm responsible for a constellation of signs, symptoms, and laboratory changes. Primary HLH typically presents in the first year of life with or without a positive family history. There is poor information regarding the relationship between HLH and the downregulation of the PI3K/AKT/mTOR signaling pathway. Mutation in the PIK3CD gene (NM_005026.3) is associated with activated PI3K delta syndrome, which is a primary immunodeficiency that could cause HLH (9). Secondary HLH is associated with infections, particularly Herpes viruses, malignancies, and autoimmune disorders. Secondary HLH can occur in children or adults. The exact mechanism of HLH secondary to a solid tumor has yet to be explained, but it is assumed that the hyperinflammation is triggered by the secretion of proinflammatory cytokines and persistent antigen stimulation by tumor cells. When HLH arises in association with rheumatologic disease, it is termed macrophage activation syndrome (MAS). MAS can be found with idiopathic arthritis and systemic lupus erythematosus, but it was also described in other rheumatological conditions (10–12). HLH associated with solid cancer is rare. In a study of 2,197 adults with HLH, only 1.46% (*n* = 32) of patients had HLH triggered by a solid tumor while it appeared in 981 patients who had hematologic malignancies (45%) (13).

To our knowledge, there are only two prior published studies of HLH triggered by gastric cancer in three patients. Our aim is to describe the fourth case in the world, and the first in Western countries, and make a brief review on HLH in gastric cancer precisely because HLH is a rare condition and is little known to oncologists.

## Case presentation

A 50-year-old man went to the emergency room (ER) for hyperchromic urine, melena, and gingivorrhagia. A blood count showed Hb 14.1 g/dl (12–18), white blood cells 6.83×10^9^/L, platelets 29×10^9^/L (150–450), haptoglobin 0.4 g/L (0.3–2.0), fibrinogen 57 mg/dl (150–450), INR 1.68 (0.8–1.2), D dimer 35,000 µg/L (<500), reticlulocytes 1.97% (0.5–2.5), total bilirubin 0.73 mg/dl (0.2–1.3), PCR 7.53 mg/L (<3.5), creatinine 0.88 mg/dl (0.7–1.2), and AST 65 IU/L (8–47). The patient had no fever, and blood pressure and oxygenation parameters were normal; he complained of chest pain and had spontaneous abdominal wall hematomas. In his medical history, the patient reported arterial hypertension and myocardial infarction 3 years earlier with coronary stent placement. The cardiological assessment with an electrocardiogram showed outcomes of antero-septal necrosis and troponin was 0.034 mg/ml (<0.07). Faced with this laboratory picture, the 100-mg acetylsalicylic acid that he took daily was suspended. The patient was taking 3.75 mg of bisoprolol daily; 7.5 mg of zofenofril calcium, one tablet in the morning and one tablet in the evening; and 20 mg of atorvastatin daily. On the basis of the first tests, the hematologist diagnosed disseminated intravascular coagulation (DIC) with an ISTH score of 6 and a PLASMIC score of 3. A CT scan of the chest and abdomen with a contrast agent showed lymph nodes redundant in number and size in the mediastinum, in the celiac area, in the hepatic peduncle, and in the lumbar aortic retroperitoneum with a short axis of 12 mm. The bone window described some millimetric areoles of an osteorarefactive aspect in some dorsolumbar vertebrae and on the posterior arch of the fifth rib.

The bladder was normally distended with homogeneous content. A blood smear examination revealed rare schistocytes and some neutrophils with nucleus hypersegmentation. In the absence of fever, “cold” blood cultures were performed to rule out infection and subsequently yielded negative results. In the ER, the patient received a bag of platelets, and subsequently, in the Department of Medicine, he received 24 bags of fresh plasma in the first 2 days in addition to undergoing plasmapheresis. Prophylactic therapy started with meropenem 1 g three times a day. After 7 days of hospitalization, a PET-CT scan showed areas of pathological increase in glucose consumption on the lymph nodes of the mediastinum in the left paratracheal, subcarinal, and in the Barety lodge (SUV 8.3), in the celiac plexus and bilateral lumbar-aortic (SUV 8), and in the right lateral cervical (SUV 14.3), as well as multiple and disseminated areas of focal pathological accumulation in the medullary compartment of all skeletal segments (SUV 15.6). On the eighth day, a bone marrow aspirate was performed with subsequent evidence of numerous images of hemophagocytosis. A subsequent bone marrow aspiration demonstrated the absence of blast cells, karyotype abnormalities, and rearrangements of RAR and ABL1. The patient meanwhile received transfusions of fresh plasma daily and a bag of platelets almost every 2 days in the presence of severe thrombocytopenia. On the eighth day, a series of blood tests were performed: HIV 1 and 2 negative, IgM (negative) and IgG (positive) for Herpes simplex, IgM (negative) and IgG (positive) for Toxoplasma, and IgM (negative) and IgG (positive) for Epstein–Barr virus (EBV). Lupus anticoagulants (LAC) 1 and 2 were high with a LAC1/LAC2 ratio of 1.12 (<1.3), anticardiolipin antibodies (IgG and IgM) were negative, neutrophil anti-cytoplasmic antibodies (MPO and PR3) were negative, antibodies to B2 glycoprotein (IgM and IgG) were negative, the reuma test was <10 UI/ml (<15.0), complement C3 was 1.11 g/L (0.9–1.8) and C4 was 0.11 g/L (0.1–0.4), and a Widal–Wright test was negative.

On the ninth day of hospitalization, platelets had dropped to 15×10^9^/L, Hb was 6.19 g/dl, and white blood cells were 7.57×10^9^/L. With the suspicion of an immune-related cytopenia and DIC, the patient received oral etoposide 100 mg twice daily for 5 days, intravenous immunoglobulin 1 g/kg for 3 days, and intravenous methylprednisolone 1 g daily for 5 days. During hospitalization, the patient underwent transfusions of fresh plasma almost daily, a platelet transfusion was administered every 2 or 3 days, and a blood transfusion was administered a little less frequently.

On the 17th day, a supraclavicular lymph node was removed with a diagnosis of lymph node metastasis of adenocarcinoma compatible with gastric origin. By immunohistochemistry, neoplastic cells were positive for cytokeratin 7 and CDX2, and negative for CK20, TTF1, napsin A, PAX8, CD30, PLAP, and S100.

On the 23rd day, a fever of up to 38°C appeared, blood cultures were performed, and treatment was started with 400 mg of teicoplanin and 2 g of ceftazidime three times daily and 500 mg of metronidazole three times daily for 6 days. The blood cultures were negative and the fever disappeared within 7 days. On the 27th day, gastroscopy revealed, in the antrum, body, and fundus, a mucous membrane with a cobbled appearance. A biopsy was positive for infiltrating gastric carcinoma G 3 with signet ring cells.

**Figure 1:**
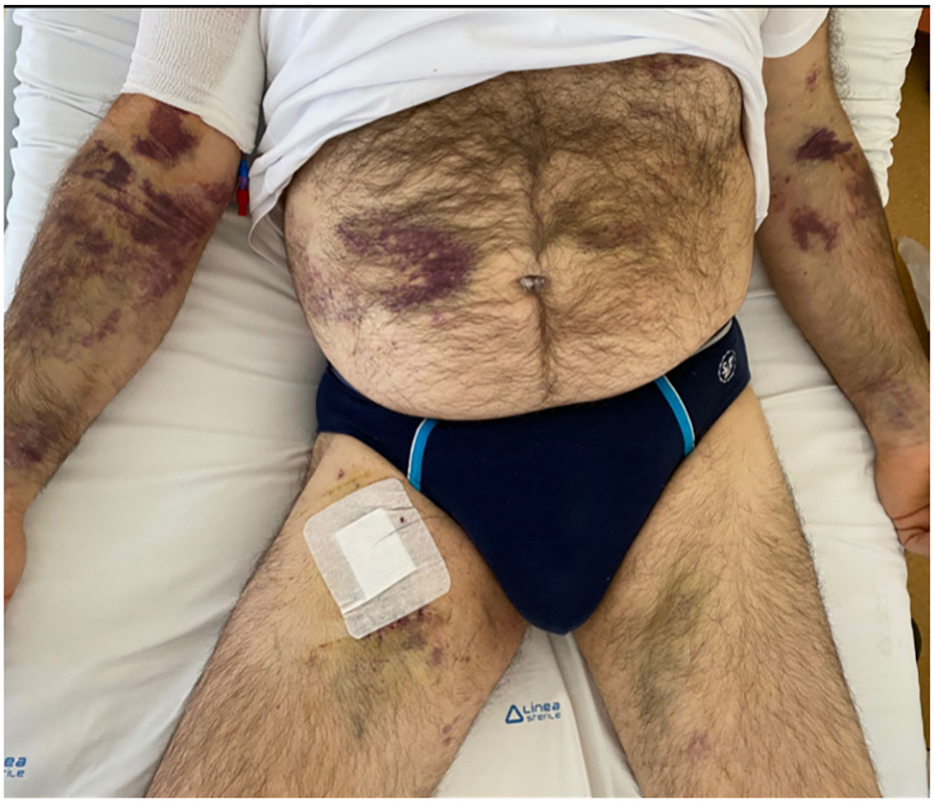
Cutaneous hematomas on oncology ward admission.

On the 30th day, the patient was transferred, at his request, to our oncological institute—a referral center far from his residence. The entrance examinations showed plt 13×10^9^/L, white blood cells 5.93×10^9^/L, Hb 9.4 g/dl, fibrinogen 228 mg/dl, INR 1.5, total bilirubin 1.55 mg/dl, direct bilirubin 0.62 mg/dl, triglycerides 274 mg/dl (<150), and ferritin 15,026 µg/L (30–400). The physical examination was unremarkable except for diffuse cutaneous hematomas (**Figure 1**), and he had asthenia with a poor performance status. He was supported with a platelet transfusion, underwent a bone biopsy, and started a chemotherapy treatment with 85 mg/m^2^ of oxaliplatin on day 1, 200 mg/m^2^ of calcium levofolinate on day 1, 400 mg/m^2^ of 5-fluorouracil bolus on day 1, 2,400 mg/m^2^ of 5-fluorouracil on day 1 for 48 h (mFOLFOX6), and 16 mg of dexamethasone daily equal to 80 mg of methylprednisolone calculated on the basis of 1 mg/kg (for 39 days then tapering off by halving the dose every week until discontinued). The bone biopsy showed a picture compatible with myelophthisis from diffused medullary localization of a carcinoma of gastric origin. The biopsy was composed of extensive necrotic material without any viable neoplastic cells. Through immunohistochemistry, necrotic cells were found to be diffusely reactive for cytokeratin 7 (**Supplementary Figure 1**). A second cycle of mFOLFOX was repeated, by timing, after 2 weeks while the third cycle needed a few days’ postponement due to neutropenia (0.7×10^9^/L). During hospitalization in the oncology ward, the patient continued to have low platelet values with the need for a platelet transfusion almost every other day and a moderate need for a blood transfusion. Unlike hospitalization in the medicine ward where a plasma transfusion was preferred for low levels of fibrinogen, recombinant fibrinogen (Haemocomplettant^®^) 1 g e.v. was used in oncology if fibrinogen was <100 mg/dl. The patient received 12 bottles of Haemocomplettant during the 42 days of hospitalization in the oncology ward. Six days after the third cycle of mFOLFOX6, the patient was discharged with white blood cells 7.47×10^9^/L, platelets 25×10^9^/L, Hb 10.3 g/dl, fibrinogen 133 mg/dl, and INR 1.14. The patient continued the chemotherapy in Day Hospital with an improvement in his general condition. In August 2021, after four cycles of chemotherapy, a total body CT scan showed a stable disease, and the scan with bone window showed the appearance of widespread osteostructural inhomogeneity with a prevalent thickening character in all bone segments as per probable response to therapy.

**Figure 2:**
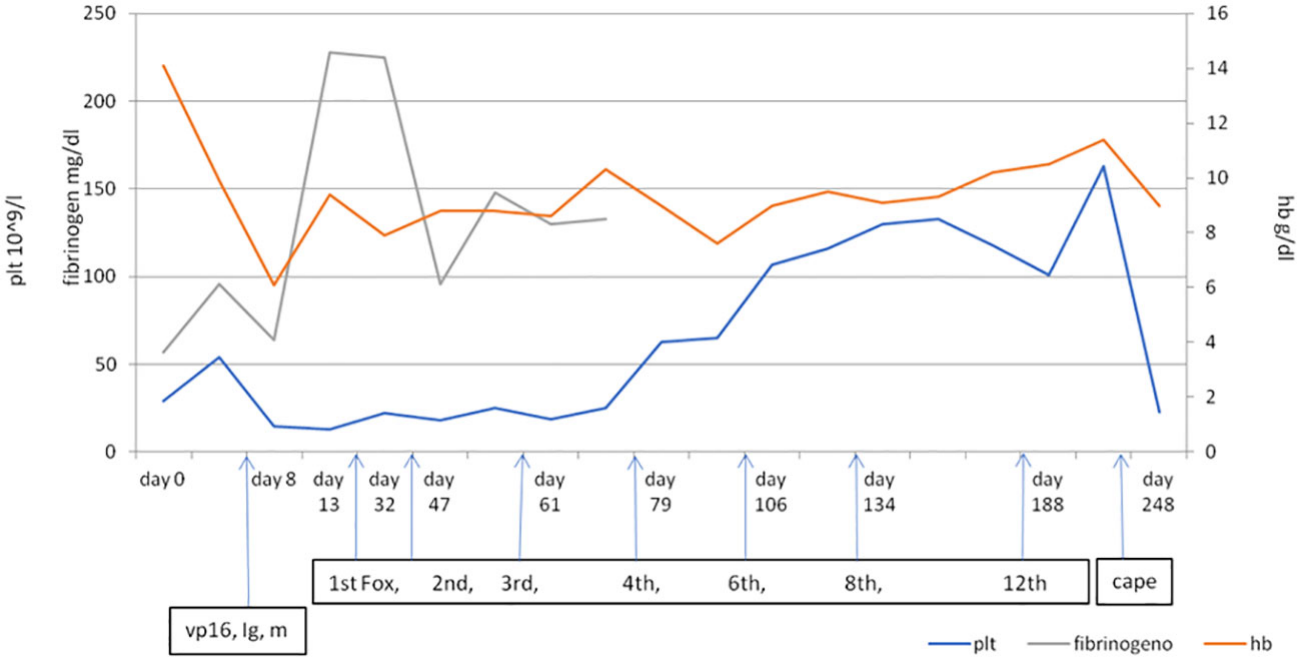
The trend of platelets, hemoglobin, and fibrinogen in relation to the treatments. Vp16, etoposide; Ig, immunoglobulin; m, methylprednisolone. First, 2nd, 3rd, 4th, 6th, 8th, and 12th cycles of Fox (Folfox); cape, capecitabine.

After numerous cycles of FOLFOX chemotherapy, up to the 12th cycle, there was an improvement in platelet (101×10^9^/L) and hemoglobin (10.5 g/dl) values. In December 2021, after 12 cycles of FOLFOX, a new total body CT scan showed slightly reduced lymphadenopathies both at the mediastinal level and at the intra- and retroperitoneal level. The scan with bone window showed an increase in osteostructural subversion with a prevalent thickening character in all bone segments due to an increase in sclerosis (**Supplementary Figure 2**). In light of the CT picture, because of the improved blood values, it was decided to start maintenance chemotherapy with capecitabine 1,250 mg/m^2^ twice daily on days 1–14 of a 3-week cycle, but after only one course of therapy, the patient went to the local ER of his hospital with vomiting and hematomas at the level of the abdominal wall. A CT scan showed a small cerebral hemorrhage, platelets were 23×10^9^/L, and Hb level was 9 g/dl. The patient was hospitalized and died a few days later. **Figure 2** shows the trend of platelets, hemoglobin, and fibrinogen before and after treatment.

## Discussion

Secondary HLH is a reactive mononuclear phagocytic response that occurs in association with a constellation of conditions such as malignancies and infections. The increased macrophage activation and macrophage proliferation produce upregulation of ferritin transport/secretion and inhibition of lipoprotein lipase and then an increase in serum ferritin and serum triglycerides, respectively. The increase in phagocytic activity provokes cytopenia too. Solid tumors associated with HLH are rare, and subsequently, an early diagnosis is more difficult. It is essential in this kind of syndrome to make an early diagnosis because without treatment, HLH is frequently fatal. In secondary HLH, 1-month mortality is estimated at 20% (14) and the median overall survival among patients with an underlying malignancy is 1.4 months (15), so prompt recognition is essential. Recently, we have had to consider another cause of HLH because immunotherapeutic strategies, used to treat several malignancies, may connect to this syndrome as well (16, 17).

Furthermore, we have to consider that HLH can occur not only during the development but also during the recurrence or relapse of malignancy (18). Various studies are trying to shed light on adult HLH.

In 2004, the Histiocyte Society proposed an updated set of criteria (HLH 2004 diagnostic criteria) to aid in the identification of patients with HLH for clinical trials (19). To make an HLH diagnosis, five of the following eight criteria must be met: fever, splenomegaly, cytopenias affecting ≥2 lineages (hemoglobin <9 g/dl, platelets <100×10^9^/L, and neutrophils <1.0×10^9^/L), hypertriglyceridemia (≥265 mg/dl) and/or hypofibrinogenemia (≤150 mg/dl), hemophagocytosis (in bone marrow, spleen, or lymph node), hyperferritinemia (≥500 µg/L), impaired NK cell function, and elevated soluble CD25 (sCD25) (i.e., sIL2R) (≥2,400 U/ml). sCD25 is not readily available at all institutions and can take time to return. It is important to note that these criteria were developed for the diagnosis of primary HLH. There is currently no universally accepted set of criteria for diagnosing HLH in the adult population.

In 2014, standardized criteria for the diagnosis of secondary HLH were published. Fardet et al. created and validated the HScore, which includes nine weighted variables (20). In our case, if we consider this scoring system available online (http://saintantoine.aphp.fr/score/), the probability for it to have been HLH is about 89% [maximal temperature strictly less 38.4°C, lower hemoglobin level less than or equal to 9.2 g/dl, higher ferritin level (ng/ml) strictly greater than 6,000, higher triglyceride level (mmol/L) between 1.5 and 4, lower fibrinogen level (g/L) less than or equal to 2.5, higher SGOT/ASAT level (UI/L) greater than or equal to 30, and hemophagocytosis features on bone marrow aspirate]. A web-based Delphi study proposed a similar list of criteria (21). These newly proposed criteria are a step towards improving the diagnosis of HLH in adults. The first obstacle or rather the first challenge for the clinician is, therefore, to try to understand an atypical clinical picture.

In our case, the first diagnosis was DIC, and then on the eighth day from admission to the ER, a diagnosis of HLH was hypothesized based on the low value of platelets, hemoglobin, and fibrinogen, and the description of numerous images of hemophagocytosis in the bone marrow aspirate; however, a fifth element was not looked for. The clinicians had no initial information about hypertriglyceridemia, low or absent natural killer cell activity, ferritin, increased soluble CD25 concentration, and splenomegaly while the fever appeared. It is interesting to note that DIC is reported in 40% of the cases in some series of HLH and is associated with high mortality rates, especially in patients with severe thrombocytopenia (13).

There is a lack of relevant trials to guide the treatment of cancer patients with HLH. The immunosuppressive agents used in the treatment of HLH mainly include glucocorticoids, cyclophosphamide, cyclosporine, etoposide, and doxorubicin (19, 22).

Treatment in adults has been based on the HLH-94 study, a large prospective pediatric study conducted by the Histiocyte Society in 16-year-old patients with no history of immunosuppression or malignancy. This study investigated a treatment regimen that included an 8-week induction with dexamethasone and etoposide. If neurologic symptoms were present, they also received intrathecal methotrexate. If familial disease or relapsing disease was present, the patient received maintenance therapy with dexamethasone pulses, cyclosporine daily, and etoposide until the patient was able to undergo a stem cell transplant (23). This regimen resulted in improved outcomes but there were a significant number of early relapses (24).

As shown in **Figure 2**, it appears that the level of fibrinogen became more stable starting from mFOLFOX but probably also with the use of the Haemocomplettant rather than fresh plasma. In the same way, the trend of platelets and blood seems to become more stable with the reduction of dependence on blood transfusions and blood products. The calculation of the consumption of blood, platelets, and fresh plasma (**Supplementary Figure 3**) is based on three different time periods, and this could affect the value of this calculation and could be related to the management of different equipment.

To our knowledge, this is the fourth case of HLH in gastric cancer and the first case in a Western country. In 2021, Ramkumar, from India, reported a case of HLH in a 53-year-old man who underwent gastric endoscopic biopsy due to anorexia, persistent vomiting, and progressive mild pancytopenia (25). The patient underwent fine-needle aspiration cytology (FNAC) for supraclavicular lymphadenopathy, which showed lymph node histiocytosis and hemophagocytosis in the lymph node aspirate. The diagnosis was signet ring cell carcinoma of the stomach. A bone marrow aspiration biopsy revealed mild trilineage marrow hypoplasia with occasional hemophagocytic foci. The HLH preoperative diagnostic workup revealed hyperferritinemia (910 ng/ml), hypertriglyceridemia (490 mg/dl), and increased serum interleukin (IL-2) levels. The patient tested negative for EBV; hepatitis A, B, C, and E viruses; Widal; malarial parasite; and dengue. He underwent distal gastrectomy and a relative improvement of blood count was first observed on the first postoperative day (total count 3.9 × 10^9^/L, platelet count 120 × 10^9^/L, and Hb 10.5 mg/dl), but 2 weeks after surgery, gastrointestinal bleeding appeared and new tests were performed that confirmed HLH recurrence.

Again, in 2021, Zhou et al. from China (26), presented two cases of gastric cancer complicated with HLH. The patients received treatment including immunosuppressive agents immediately. After therapy, the two patients showed partial remission, but both died due to HLH relapse or progression of the primary tumor. The first was a 68-year-old man with intermittent fever and anorexia. He did a gastroscopy, and it revealed a tumor at the antrum of the stomach. Postoperative pathology confirmed gastric cancer, which was staged as pT1aN0M0 according to the 8th edition of the American Joint Committee on Cancer staging manual. After 1 week, the patient developed a fever but blood tests showed no evidence of hepatitis, human immunodeficiency virus (HIV), EBV, cytomegalovirus (CMV), syphilis, tuberculosis, bacterial infection, or other complications. A bone marrow biopsy indicated hemophagocytosis. After two cycles of the chemotherapy (20 mg of liposomal doxorubicin for 1 day, 100 mg of etoposide for 1 day, and 20 mg of methylprednisolone, twice daily for 3 days), the patient remained disease-free for 5 months but died from HLH relapse. The second patient was a 54-year-old woman with unresectable gastric adenocarcinoma diagnosed 5 months previously. After six cycles of chemotherapy, the patient was admitted to the hospital due to an unexplained fever lasting 10 days. She had hemorrhagic rashes all over her body and petechiae in her mouth. Blood tests indicated anemia, hypothrombinemia, and hypofibrinogenemia, as well as a significant increase in the levels of ferritin and sCD25. A bone marrow biopsy indicated hemophagocytosis. Secondary HLH was confirmed, and chemotherapy, based on etoposide (200 mg twice a week) together with dexamethasone (20 mg daily), was subsequently administered. She had an initial clinical benefit, rather than a laboratory benefit, and received three cycles of chemotherapy. Considering the pancytopenia, cyclosporine (75 mg twice daily) was continuously administered, but chemotherapy was stopped. The patient remained in a stable condition for 8 weeks, then intestinal bleeding appeared with subsequent renal failure and multiple organ failure.

In **Table 1**, we report a comparison of a diagnosis with three other cases of secondary HLH in gastric cancer patients. In the case reported by Ramkumar (25) and in our case, the diagnosis was for a signet ring cell cancer, while we have no clear information about the histology of gastric cancer in the other two cases by Zhou (26). Thus, we cannot speculate about the risks of HLH related to histology. In the case from Ramkumar (25) and in one case from Zhou (26), the HLH “exploded” in a clinical manner after a gastrectomy; if one does not know about HLH, it seems strange to learn of deaths after gastric cancer resection. In our case, the patient had the metastatic disease as in the case presented by Ramkumar (supraclavicular lymphadenopathy), while in one case presented by Zhou, the patient had unresectable gastric adenocarcinoma. HLH is likely to underlie the expression of an advanced gastric carcinoma. Gastric cancer most commonly spreads to the peritoneum, liver, or lungs while the incidence of bone metastasis is 2%–3% (27). Bone metastases diffusely invading the bone marrow from gastric cancer often manifest a rapid clinical course, and the prognosis is very poor due to hematologic disorders such as DIC and/or MAHA (microangiopathic hemolytic anemia). Etoh et al. (28) described that the median survival time in bone metastasis from gastric cancer was 2 and 11 months for the patients with or without hematologic (DIC and/or MAHA) disorders, respectively. Both disseminated DIC and thrombotic microangiopathy (TMA) cause microvascular thrombosis associated with thrombocytopenia, bleeding tendency, and organ failure (29). MAHA is required in TMA. DIC is often associated with TMA, and TMA is often associated with DIC, suggesting that a differential diagnosis between DIC and TMA may be difficult. In this report, clinicians tried to make a differential diagnosis using ISTH (score, 6), PLASMIC (score, 3), and HLH 2004 diagnostic criteria. The ISTH (score, 6) showed laboratory evidence consistent with DIC, and the PLASMIC score was used to stratify patients into low (0–4), intermediate (5), and high (6–7) risk of thrombotic thrombocytopenic purpura. The HLH 2004 diagnostic criteria evidenced five out of eight diagnostic criteria. Ultimately, in our case of HLH, there is also a DIC, but as we have tried to summarize above, there are hematological clinical pictures that can intersect and complicate the diagnosis. A limitation of our case report could be that hemophagocytosis was described from bone marrow aspirate while the bone biopsy did not show hemophagocytosis but infiltration by tumor cells; however, it is plausible that the initial therapy with etoposide, immunoglobulin, and steroid partially modified the HLH substrate. A second limitation is that we have no information about NK cell function, and sCD25. A third limitation is that we do not have clear data about the last admission of the patient to the ER at his local hospital, but his daughter informed us about her father’s skin hematomas and small cerebral hemorrhage associated with piastrinopenia. An interesting aspect of our case is the good laboratory and clinical response obtained with FOLFOX chemotherapy and also the discrete survival of about 8 months. This survival, although limited, should be considered a good result for a patient with bone metastases from gastric cancer and, above all, with coagulation alteration if compared with literature data (28).

**Table 1:** Cases and hemophagocytic lymphohistiocytosis (HLH) 2004 diagnostic criteria.

HLH 2004	Case 1 Ramkumar	Case 2 Zhou	Case 3 Zhou	Case 4 Monti
Fever 38.5°C or higher		+	+	
Splenomegaly	+	+		
Cytopenia (affecting at least two of three cell lineages in peripheral blood)	+	+	+	+
Hypertriglyceridemia and/or hypofibrinogenemia	+		+	++
Hemophagocytosis in bone marrow or spleen or lymph nodes	+	+	+	+*
Low or absent NK cell activity		+		
Ferritin ≥500 μg/L	+	+	+	+
sCD25 ≥2,400 U/ml	+	+	+	

*Hemophagocytosis was present on the bone marrow aspirate.

In this report, we showed a challenging case where secondary HLH is associated with gastric cancer. The oncologist has to keep in mind the existence of HLH when there is an unusual clinical presentation of cancer disease like cytopenias affecting ≥2 lineages and consider other pathophysiological possibilities besides myelophthisis and DIC.

Our experience points out the importance of finding and treating the cause of HLH as soon as possible, and low blood counts should not discourage the oncologist from treating the patient with chemotherapy in the same way as with hematological patients with bone marrow involvement.

## Data availability statement

The raw data supporting the conclusions of this article will be made available by the authors, without undue reservation.

## Ethics statement

Ethical review and approval was not required for the study on human participants in accordance with the local legislation and institutional requirements. The patients/participants provided their written informed consent to participate in this study. Written informed consent was obtained from the individual(s) for the publication of any potentially identifiable images or data included in this article.

## Author contributions

MM and GM designed the study; CG and VG did the literature search; LE, SA, DM, VG, and GF collected and analyzed the data. AA-S performed the pathological diagnosis of the bone marrow biopsy; MM wrote the manuscript; all authors contributed to the interpretation of the data; all authors read and approved the final version of the manuscript.
