# Supplementary material for: Hemophagocytic lymphohistiocytosis in gastric cancer: A rare syndrome for the oncologist. Case report and brief review
Source: Front Oncol. 2023 Feb 8;13:1010561. doi: 10.3389/fonc.2023.1010561 (PMC9945267; doi:10.3389/fonc.2023.1010561)
Supplement: Supplementary file 1 [file DataSheet_1.pdf]

frontiers

Supplementary Material

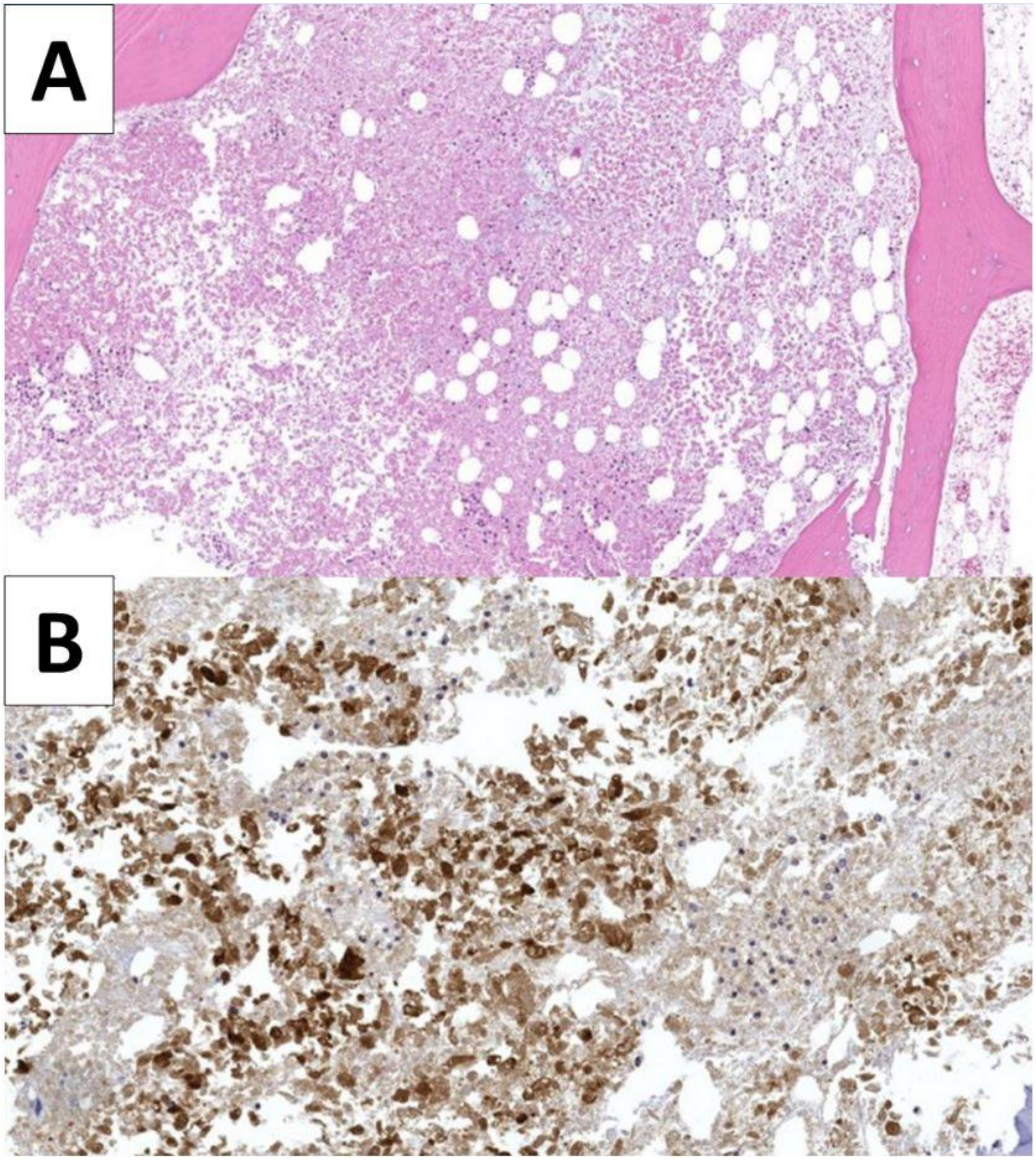

Supplementary Figure 1. Bone marrow biopsy showed extensive necrotic material (**A**: low power) with rare inflammatory cells. By immunohistochemistry, necrotic cells were diffusely immunoreactive for cytokeratin 7 (**B**: low power). Considering the previous history, these features were consistent with bone marrow metastasis from gastric carcinoma.

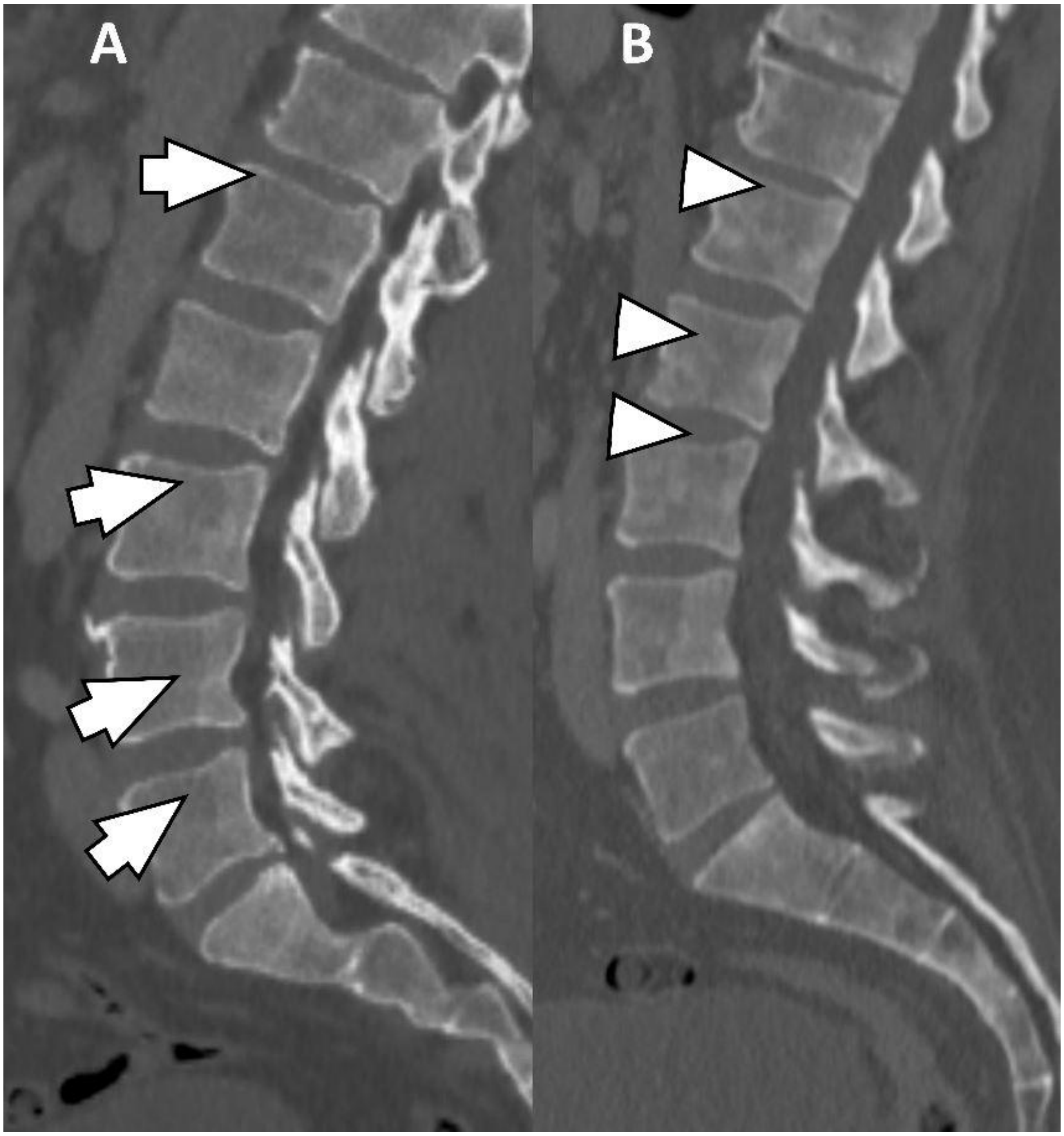

Supplementary Figure 2. (**A**) The baseline total body contrast enhanced CT scan showed several enlarged lymph nodes in the mediastinum, in the celiac area, in the hepatic peduncle and in the lumbar aortic retroperitoneum. Additionally were described few osteorarefactive areas (white arrows) in some dorsolumbar vertebral bodies. (**B**) The subsequent total body contrast enhanced CT scan showed slight reduction of the thoracic and abdominal lymphadenopathies. Additionally, an increase of the sclerotic component of the bone lesions (white arrowheads) was noted.

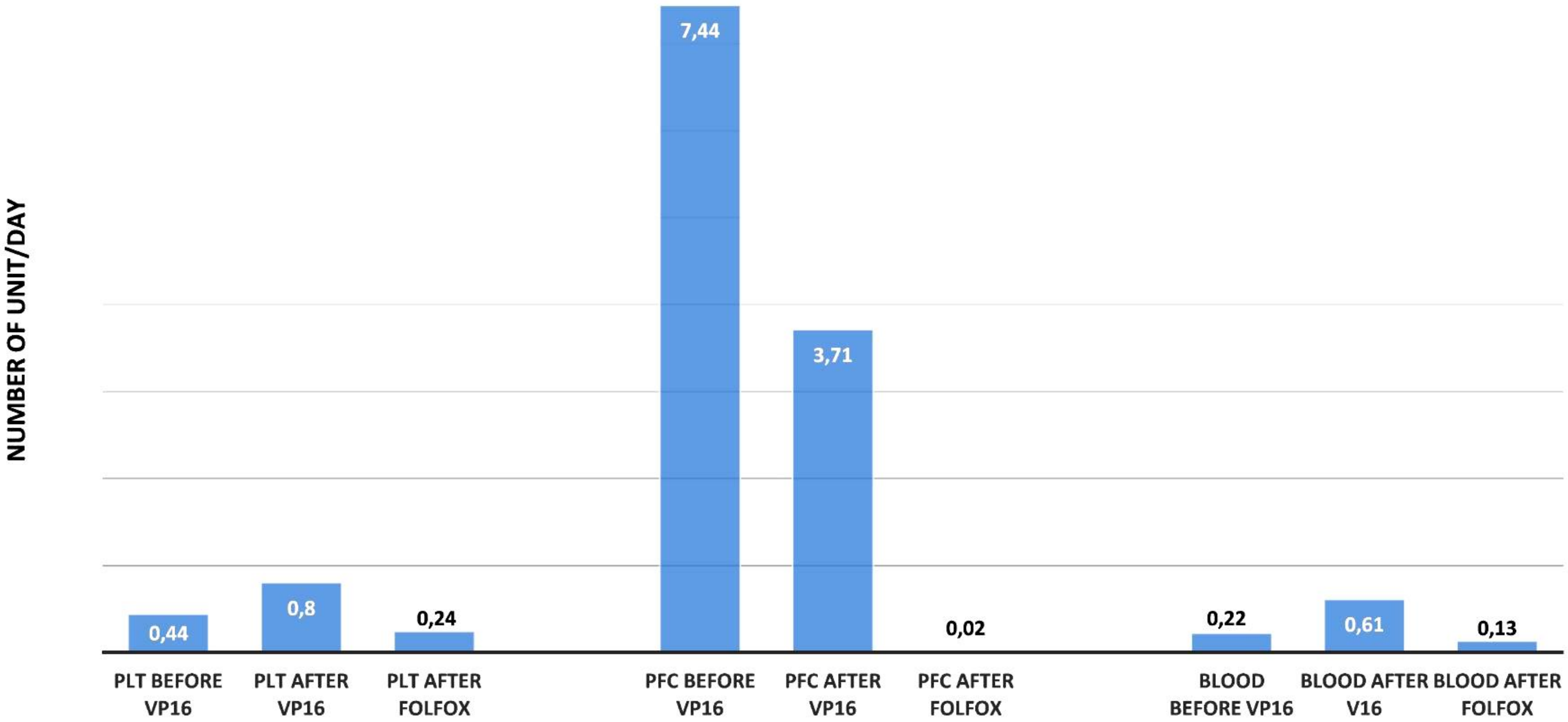

Supplementary Figure 3. Consumption of blood components per day. The calculation is obtained by dividing the total units (of platelets or blood or fresh plasma) by the number of days elapsed between access to the ER and the start day of vp16, or between the day following the start of vp16 and the day of the 1st mFOLFOX, the day following the start of the 1st mFOLFOX and discharge.
